# Significant Impact of Schmallenberg Virus in Three Ruminant Farms: A Laboratory Experience

**DOI:** 10.1155/vmi/9979035

**Published:** 2026-01-05

**Authors:** Jacopo Guccione, Valentina Longobardi, Maria Chiara Alterisio, Ugo Pagnini, Gianmarco Ferrara

**Affiliations:** ^1^ Department of Veterinary Medicine and Animal Production, University of Naples Federico II, Naples, Italy, unina.it; ^2^ Department of Veterinary Sciences, University of Messina, Messina, Italy, unime.it

**Keywords:** animal selection, arbovirus, embryo transfer, impact, SBV, Schmallenberg virus

## Abstract

Schmallenberg virus (SBV) is endemic in Europe and other parts of the world and represents an underestimated and underdiagnosed cause of abortion and economic losses for livestock farms. In the present study, we evaluated SBV’s impact on three ruminant farms, in particular, two dairy cattle farms with reproductive problems (including abortions, stillbirths, and malformations) and a clinically healthy buffalo farm involved in oocyte collection through ovum pick‐up (OPU) for in vitro embryo production and commercial sale. All sampled animals were subjected to serological assays against the main infectious agents responsible for reproductive disorders in ruminants: SBV, bluetongue virus (BTV), *Coxiella burnetii* (*C. burnetii*), bovine herpesvirus type 1 (BHV‐1), bovine viral diarrhea virus (BVDV), and *Brucella abortus* (*B. abortus*). The first herd with reproductive disorders had a high (78%) seroprevalence for SBV (considered to be the cause of reproductive problems), while the prevalence observed for BTV was modest (16.7%). Although free from *B. abortus*, the second dairy farm had only a few animals that were seropositive for *C. burnetii* and BTV (1/22 and 2/22, respectively), but a prevalence of 72.7% for SBV. The buffalo farm had several animals seropositive for *C. burnetii* (9/52), BTV (10/52), and SBV (12/52). Almost half of the sampled animals were exposed to at least one pathogen (26/52). Seropositive animals were excluded from oocyte collection in compliance with regulatory health requirements. One month later, seronegative animals were retested, revealing the seroconversion of another animal for SBV, which was also excluded. This study has described, through seroprevalence testing, the direct impact of SBV on livestock (clinical impact) and how it affects the selection of animals for the trade of genetic material (indirect impact).

## 1. Introduction

Schmallenberg virus (SBV) is an RNA arbovirus that infects mammals and has a clinical impact on ruminants [1]. This peribunyavirus is transmitted from animal to animal through the bite of blood‐sucking insects of the *Culicoides* genus, and due to climate changes that are affecting the whole planet, it is now widespread in much of the world and in most months of the year [2–4].

The clinical impact of this infection is characterized by mild diarrhea and reduced milk production (due to fever), as well as reproductive disorders involving the fetus: abortion and mummification or fetal malformations, such as arthrogryposis and hydrocephalus [5, 6]. Due to the clinical symptoms described above, the economic impact of this infection is significant and even more so in endemic situations. The infection appeared in Europe in 2012 and has spread to several European countries, including those in Northern Europe, following the spread of its vector [7, 8]. SBV, although endemic today, manifests itself symptomatically with fairly regular cycles that are influenced by the duration of active immunity and the degree of exposure of the animals to the virus (and therefore the degree of protection against reinfection) [2, 9, 10]. In fact, after its discovery, SBV has been responsible for cyclical outbreaks over the years [11, 12]. This cyclicity has affected susceptibility, circulation, and therefore the seroprevalence observed in different studies. For example, in Germany, prevalence was approximately 5% in 2022 and increased to about 32% in 2023. Similar scenarios also occurred in Belgium, Poland, and Ireland [13–16]. When there are no outbreak conditions but only endemicity, only a small number of abortions are attributable to SBV. This was highlighted in a Hungarian study which detected at least one infectious agent in 537 abortions, in which SBV infection was confirmed in only 0.8% of them [17].

Several studies have described the impact of SBV on ruminant livestock. Evidence from several countries has demonstrated that this virus is capable of causing losses ranging from 25 to 50 euros per cow/year and an additional 1700 to 2000 euros per farm/year depending on the type of outbreak (low‐ and high‐impact scenarios) [18–21].

These financial consequences range from 0.6% to 63% of the gross margin, depending on the situation and livestock system under consideration. SBV’s effects are mostly caused by the additional expenses associated with acquiring and raising replacement heifers, as well as losses in milk yield (dairy cows), losses in calf or lamb production, and losses in milk production and unsold replacement lambs [18, 20, 21]. These different investigations demonstrated how the combination of production and financial models may be used to estimate disease effects in the dairy sector while accounting for differences in management and husbandry procedures, as well as costs.

In Southern Italy, the circulation of the virus was reported only recently, but it has been assumed that the virus was present in these districts already with the first European outbreaks [22, 23]. A recent study highlighted a seroprevalence of 40.5% higher in young animals and those coming from districts with climatic characteristics favorable to the *Culicoides* cycle [22].

This study describes the serological status related to selected abortigenic agents of three farms (two cattle farms and one dairy buffalo farm) where SBV circulation was detected. The aim was to highlight the exposure to SBV and other abortigenic agents on ruminant farms with different production systems in Southern Italy.

## 2. Materials and Methods

### 2.1. General Conditions Regarding Sampling

This study was carried out between 2021 and 2022 and includes data from three farms in Southern Italy that requested diagnostic assistance from the Department of Veterinary Medicine and Animal Production of Naples (DVMAP) to explore the reasons for abortion or performing surveillance in their herd. The farms were chosen using a convenience sampling (i.e., only those that approached the department for diagnostic assistance within the specified study period were included). A representative sample method was used based on farm size (at least 10%, with the aim of including any animals that had experienced reproductive problems).

### 2.2. Farm Study *n*1 and Sampling

**Figure 1:**
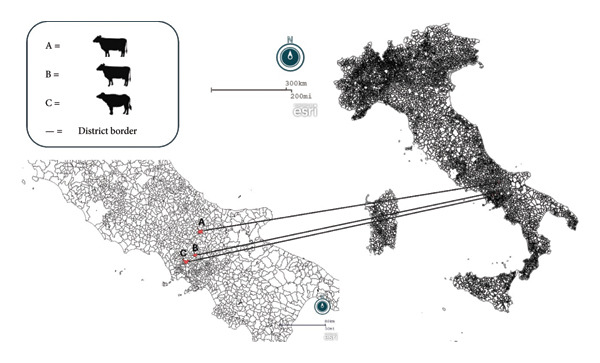
Representation of the sampled farms created using the geographic information system Epi Info (https://www.cdc.gov/epiinfo/index.html). A, B, and C represented the location of the sampled farms: two cattle farms (A and B) and one water buffalo farm (C). The upper scale bar shows a 300 km (200 mile) segment and the lower one 80 km (50 miles).

Farm number 1 was in Campobasso, in Southern Italy, an area where dairy cattle breeding is widespread (Figure 1). The case was referred to the Ambulatory Clinics Service of the Veterinary Teaching Hospital of the DVMAP in July 2021. The farm, which carried out internal replacements, had 170 animals (Friesian), of which approximately 85 were lactating. Heifers were vaccinated before giving birth against rotavirus, coronavirus, and *Escherichia coli* K99 (Bovilis Rotavec Corona, Bovilis). After the fourth week and the sixth month of life, calves receive an anti‐bovine viral diarrhea virus (anti‐BVDV) vaccination (Bovilis BVD). The herd is officially *Brucella abortus* (*B. abortus*) and bovine herpesvirus type 1 (BHV‐1) free. The farmer was concerned about an increase in the stillbirth/perinatal mortality [24] and an increase in the average number of services per pregnancy (2.34). Furthermore, recent cases (April–July 2021) of hydrocephalus and arthrogryposis have been described, especially in heifers. Given the suspicion of an abortigenic infectious disease, a representative sample of 8 calves and 10 adults was collected, including a calf with arthrogryposis that survived (defined as deformed angulation of limbs due to persistent flexion/fusion of the joints), and the cows that had stillbirth/perinatal mortality [25]. Moreover, clinically healthy animals (healthy cows or calves were defined excluding a series of physical and behavioral alterations that indicate not good health at examination time) and animals with diarrhea (feces classified as score 2 = loose feces, or 3 = watery feces according to Jaureguiberry et al. [26]) have been sampled (blood samples). All samples were sent to the laboratory of infectious diseases of domestic animals of the same department to perform specific serological investigations.

### 2.3. Farm Study *n*2 and Sampling

Farm number 2 was a dairy farm in Solopaca, in Southern Italy (Figure 1). Almost 200 animals were bred (Friesian and Italian Red Pied). In the period May–July 2022, the farmer complained of an increase in the rate of stillbirth and abortion (over 15%). The farm was officially brucellosis‐free, and a vaccination plan was adopted against BHV‐1 and BVD (HIPRABOVIS 9, HIPRA). A total of 22 blood samples (13 adults and 9 young) were collected in September 2022 and transported to DVMAP to receive the same investigations carried out for farm study 1.

### 2.4. Farm Study *n*3 and Sampling

Farm number 3 raised high‐genealogy water buffalo (*Bubalus bubalis*) in the province of Caserta, an area with the highest concentration of buffalo breeding in the country. The farm reared about 400 dairy buffalo and collaborated with a company (Embrionica s.r.l, Naples) for the sale of “in vitro–produced” buffalo embryos abroad. All the animals received a BHV‐1 marker vaccine after 3 months of life and an annual booster (Bovilis IBR Marker Live, Bovilis). According to the current legislation, all donors of oocytes/embryos must be seronegative to several abortigenic infections. Due to these provisions, the company carries out a monitoring plan with self‐inspection to proactively identify seropositive animals. A total of 52 blood samples were collected and sent to the laboratory of infectious diseases of domestic animals of the DVMAP (September 2022). All sampled animals had their last calving in 2022, with lactation numbers ranging from first to ninth. Seronegative animals were reevaluated after 21 days (October 2022).

### 2.5. Enzyme‐Linked Immunosorbent Assays (ELISAs)

Blood samples were centrifuged, and the obtained serum samples were tested for the presence of antibodies (Ab) against SBV, *Coxiella burnetii* (*C. burnetii*), BVDV, BHV‐1, and *bluetongue virus* (BTV) using specific commercial ELISAs: ID Screen Schmallenberg virus Indirect Multi‐species (IDVet), Test IDEXX Q Fever Ab (IDEXX), ID Screen BVD p80 Antibody Competition, ID Screen IBR Indirect, and ID Screen Bluetongue Competition (IDVet). Competitive ELISAs were used for BTV and SBV, while classical indirect ELISAs were used for BVDV, BHV‐1, and *C. burnetii*. All ELISAs were carried out following the instructions provided by the manufacturer, using an automated microplate washer (Sirio S, DIA LAB Services Srl) for washing and a spectrophotometer for reading the optical densities (Thermo Scientific Multiskan GO Microplate Spectrophotometer, Thermo Fisher Scientific). Test interpretation was also performed according to the manufacturer’s instructions. In addition to commercial tests, all sera were tested for the presence of Ab against *B. abortus*, using a standard rose bengal rapid seroagglutination (RSA) protocol [27]. The reaction mixture (100 μL) consisted of 50% serum and 50% antigen (Pourquier Rose Bengale Ag, IDEXX, 0.5% phenol–saline suspension of *B. abortus* biovar 1, strain 99, inactivated, stained with Rose Bengale). After mixing, the slides were placed on a mechanical rotator at 80–100 r.p.m. for 4 min and agglutination reactions were observed.

Seroprevalence was calculated by dividing the number of seropositive animals by the total number of animals. Seroprevalence was estimated for each pathogen, farm, and animal category (adults/calves and number of lactations for farm *n*3). Fisher’s exact test was used to identify any statistically significant differences between the various groups (farm, age class, and number of lactations). *p* values less than 0.05 were considered significant (MedCalc Statistical Software, Ostend, Belgium, Version 16.4.3).

## 3. Results

### 3.1. Farm Study *n*1

No *C. burnetii*, BHV‐1, or *B. abortus* Ab among the 18 animals investigated were detected (Table 1). The BVD test revealed the presence of Ab in 6 of 8 calves, indicating that two animals (25%) did not seroconvert following immunization. Only 1/10 of the animals in production exhibited Ab against BVD, most likely due to newly acquired exposure or the presence of persistent vaccine‐induced Ab. Three adult animals tested positive for BTV Ab (16.7%). SBV had the highest seroprevalence (77.8%), with 14/18 animals (8/10 adults and 6/8 calves) tested positive for SBV Ab. Furthermore, all three BTV seropositive animals were SBV‐positive (coinfection rate of 16.7%). All animals that had aborted or calved a malformed calf tested positive for anti‐SBV Ab.

**Table 1:** Seroprevalence of 5 abortigenic agents in farms with reproductive disorders.

Factor	*n*	SBV	*C. burnetii*	BTV	BVD^∗^	BHV‐1^∗^
		Positive (%)	Positive (%)	Positive (%)	Positive (%)	Positive (%)
Total	92	42 (45.6)	10 (10.8)	15 (16.3)	29 (31.5)	72 (78.3)
Farm						
1	18	14 (77.8)	0 (0)	3 (16.7)	7 (38.9)	0 (0)
2	22	16 (72.7)	1 (4.5)	2 (9)	22 (100)	22 (100)
3	52	12 (23.1)	9 (17.3)	10 (19.2)	0 (0)	50 (96.1)
Age						
Adults	75	32 (42.7)	2 (2.7)	13 (17.3)	14 (18.7)	63 (84)
Farm 1	10	8	0	3	1	0
Farm 2	13	12	1	0	13	13
Farm 3	52	12	1	10	0	50
Calves	17	10 (58.8)	0 (0)	2 (11.8)	15 (88.2)	9 (52.9)
Farm 1	8	6	0	0	6	0
Farm 2	9	4	0	2	9	9
Farm 3	0	0	0	0	0	0
Lactation 1–4	33	9	3	3	0	31
Lactation 5–9	18	3	6	7	0	18

*Note:* BHV and BVD are indicated with an asterisk because some farms were vaccinated.

### 3.2. Farm Study *n*2

In the second herd, a total seroconversion for BHV‐1 and BVD was observed in response to the vaccination. *C. burnetii* and BTV had low seroprevalence (1/22 and 2/22, respectively). Most of the animals tested positive for anti‐SBV Ab (72.7% or 16/22), which was the most widespread infection and the probable cause of the observed reproductive disorders (Table 1). Almost all adult animals (12/13) were exposed to SBV, and four of nine calves had anti‐SBV Ab. *C. burnetii*/SBV coexposure was also identified in one animal (4.5%). Out of 22 cows, only four were seronegative for SBV, BTV, and *C. burnetii*.

### 3.3. Farm Study *n*3

A total of 9 animals were exposed to *C. burnetii* (Table 1). Furthermore, 12/52 and 10/52 tested positive for SBV and BTV Ab, respectively. Some coexposures were also observed. In fact, one animal had Ab against SBV and BTV (1.9%), one animal against SBV and *C. burnetii* (1.9%), and one animal for BVD, BTV, and *C. burnetii* (1.9%). At least one cow with lactation numbers of 1 (1/10), 2 (2/8), 3 (6/14), 5 (1/8), and 9 (1/1) was seropositive for SBV. No cows in the fourth (0/4), sixth (0/4), seventh (0/2), and eighth lactations (0/1) had anti‐SBV Ab. Only 50% (26/52) of animals were negative in all tests performed. These 26 animals were reevaluated using the same tests after three weeks, and one animal, initially negative, had anti‐SBV Ab. Of the initial 52 animals, only 25 were selected for oocyte collection.

### 3.4. Overall Seroprevalence and Statistical Analysis

The total seroprevalence was 45.6% for SBV, 10.8% for *C. burnetii*, and 16.3% for BTV (Table 1). There were no positive samples for *Brucella* Ab, and vaccinated animals exhibited appropriate anti‐BVD and BHV‐1 responses. A statistically significant difference (*p* = 0.001) in SBV seroprevalence was found between farms 1 and 2 (> 70%), which had reproductive problems, and farm 3 (23.1%). On the other hand, farm 3 had a higher number of animals exposed to BTV than farms 1 and 2 (*p* = 0.01) (supporting file 1).

## 4. Discussion

Abortive infections have a substantial economic impact on farms due to a variety of factors, such as losses from unsuccessful lactation, lost calves, veterinary medications, and national eradication program measures, as well as the disease’s transmissive and/or zoonotic potential [28, 29]. Several studies have highlighted how infectious agents are among the main causes of abortion and retained placenta in ruminant species (around 42% of abortions diagnosed as infectious) [29–31]. In this study, the spread of the primary abortigenic agents, including SBV, was assessed in three farms, two of which had reproductive issues and one that used serological screening before oocyte collection. SBV infections can have significant reproductive effects, mainly including (i) increased farm rates of embryonic deaths, (ii) abortions, (iii) stillbirths, and (iv) congenital defects. All these conditions can collectively contribute to substantial economic losses for cattle farmers. On the one hand, gestation interruptions reduce calving rates and extend calving intervals, leading to increased costs due to repeated breeding, hormonal treatments, and veterinary interventions; on the other hand, the congenital malformations (e.g., arthrogryposis, scoliosis, hydrocephalus, cerebellar hypoplasia, twisted limbs, etc.) can lead to stillbirths or severe neurological impairments in live births, impairing animal welfare and increasing dystocia and culling rate in the relative dams [11, 20, 25]. In consideration of these clinical aspects, the economic impact associated with SBV infection can be considered as complex; therefore, the implementation of effective surveillance and control measures becomes a priority to mitigate these negative effects on the cattle farming economy [11].

In the two farms with reproductive problems, the high seroprevalence for SBV indicated this virus as the presumptive cause of the symptoms observed. This seroprevalence was significantly higher than that obtained in the third farm (without any reproductive issues). Moreover, the obtained seroprevalence (> 70%) was far higher than that described in seroprevalence studies in Italy (around 40%) and Europe (between 5% and 40%) [12, 22]. The difference might be attributed to the fact that seroprevalence studies are conducted on apparently healthy animals (to prevent bias); nevertheless, the current study was conducted on animals with reproductive problems and, therefore, in the context of an epidemic of abortigenic diseases. Furthermore, the number of animals seropositive for other infections (*C. burnetii*, BTV, etc.) was compatible with the prevalence observed in clinically healthy animals considering the endemicity of these infections in Italy [32–34]. Recent studies have established a seroprevalence of 11.7% for *C. burnetii* and 43.6% for BTV [32, 33]. In the farms examined, Ab against these pathogens were present in 16.7% and 9.1% for BTV and 0% and 4.5% for *C. burnetii*, respectively, in farms *n*1 and 2.

The differences in BTV and *C. burnetii* seroprevalence regarding age and number of lactations were consistent with those reported in the literature. In fact, the number of lactations and adulthood favor seropositivity to *Coxiella* and BTV, since they enhance the likelihood of transmission with matings and/or vector seasons, respectively [22, 33]. This pattern was not found for SBV, as reported in some research, where higher seroprevalences have been observed in young animals (or at least in the first year of life) due to long‐term duration of maternally derived Ab [22].

SBV, despite being widely spread throughout Europe, is poorly identified as a cause of abortion in Italy [7]. The first described case of SBV in Italy dates back to February 2012, when the virus was identified in the brain of an aborted goat in the province of Treviso, Northern Italy [35]. Subsequently, viral circulation was also reported in other regions [36]. Vector‐borne infections, such as SBV and BTV, will become increasingly widespread and have an ever‐increasing impact due to climate change [37]. Recent studies have already highlighted how the seroprevalence of SBV depends on geographical factors, such as altitude, average temperature, and distance from the coast, considering that this trend will become more critical [22, 37].

In addition to a direct impact, observed in farms *n*1 and 2, SBV has been shown to have an indirect impact. In fact, in farm n3, more than 50% of the animals (animals of high genealogy) were not selected for the export of genetic material to foreign countries, as they were seropositive to at least one reproductive pathogen. Since a positive serological result reveals exposure rather than infection (or pathogen elimination), we should question the use of serology in these scenarios. According to Mercosur Resolution MERCOSUR/GMC/RES. N. 45/14 (adopted on November 27, 2014), EU Member States that have detected SBV in their territory may continue to export bovine animals and genetic material (such as embryos and semen) only if certificates include information on SBV testing (serology) (MERCOSUR/GMC/RES. N. 45/14). The test must be negative before and after genetic material collection. These requirements can be even more stringent depending on the state in which the genetic materials are to be exported. Furthermore, during the 2012 epidemic, numerous extra‐EU‐nations implemented limitations on EU products, including restrictions on live ruminants, import restrictions, and additional certification requirements for ruminant semen and embryos [38]. This means that farms comparable to farm *n*3 would choose animals from whom to harvest genetic material based on serological status rather than zootechnical performance. In this case, the animals having the best performance may not be chosen.

Unlike other viruses, such as BVDV, BHV‐1, and BTV, whose pathogenetic mechanisms on embryos are known and the ability to be transmitted via embryo transfer has been established, research on this area is limited to males for SBV [38–41]. Numerous studies have investigated the presence of SBV RNA in the semen of various species and breeds, with often conflicting results. Although an experimental study highlighted the presence of viral RNA in bull semen after the inoculation of 10^6^ TCID50, further studies described that only a small number of bulls shed low amounts of SBV and that the method of extraction and the protocol used are critical [42, 43]. In particular, a previous study found SBV RNA in 11 of 95 SBV‐seropositive bulls [9].

The possible implantation of infected embryos raises concerns regarding the diffusion of abortigenic infectious diseases. Most of the studies conducted in vitro have described the impossibility of “recovering” an infected oocyte or embryo with the washings suggested by international guidelines provided by the International Embryo Transfer Society (IETS) [40, 44]. However, only a few studies have assessed in vivo whether implantation of an infected embryo led to birth or abortion. A study based on BTV serotype 8, in addition to having highlighted the impossibility of decontaminating oocytes and embryos with washes, demonstrated the transmission to recipient cows after embryo transfer and the necessity of vigorous application of the directives for screening of potential donors and the collected embryos [44]. Previous research performed in pigs found several viruses in oocyte donor tissue and maturation medium, but not in the generated embryos [45]. On the other hand, farmers are concerned about losing quality genetics by selecting donors through this rationale [46]. Consequently, these concerns must be addressed by implementing measures targeted at countering abortigenic agents, particularly those transmitted by arthropod vectors, which are more difficult to control. Control against vector‐borne diseases is one of the most recurring topics in scientific research [37]. To date, in the case of SBV, active and passive surveillance is envisaged, and a vaccine has been authorized in Europe, but never used in Italy. In case of suspicion of SBV infection, sampling of whole blood (for serological tests), blood with anticoagulants (for molecular tests), and collection of vectors using specific traps are used. The presence of serological evidence alone does not entail further measures. Although carried out on a limited number of farms (3) and on a limited number of animals (40 cattle and 52 buffaloes), the current study described field situations in which farmers are faced with reproductive/commercial problems.

Moreover, the present study represented further evidence of SBV circulation in the territory of Southern Italy, describing also clinical impact. Another piece of evidence supporting this hypothesis is that many juvenile animals tested positive and were thus exposed to a single vector season. Furthermore, the results of this study indicate the need to monitor these infections to try to prevent them by putting containment measures into practice.

## 5. Conclusion

The current study documented, using seroprevalence testing, the clinical and indirect effects that SBV has on different kinds of farming. Surveillance and preventive measures for abortigenic infections, particularly those transmitted by vectors, are required to mitigate this impact.

## Ethics Statement

The animal study protocol was approved by the Institutional Ethics Committee of Department of Veterinary Medicine and Animal Production (Centro Servizi Veterinari), University of Naples Federico II (PG/2022/0093419, 20 July 2022). Animal handling and blood sampling were performed following the guidelines of the guiding principles for biomedical research involving animals. All farmers gave their informed consent prior to their inclusion before they participated in the study.

## Conflicts of Interest

The authors declare no conflicts of interest.

## Author Contributions

All authors contributed to the study conception and design. Jacopo Guccione and Valentina Longobardi were involved in the sampling, reviewing, and editing of the original manuscript. Maria Chiara Alterisio was responsible for resources and visualization. Ugo Pagnini supervised the project and has participated in the review and editing of the original draft. Gianmarco Ferrara performed the laboratory experiment, wrote the manuscript including figures (first draft), and has submitted the manuscript.

## Funding

The authors declare that no funds, grants, or other support was received during the preparation of this manuscript.

## Supporting Information

Serological profile of the tested animals and statistical analysis.

## Supporting information


**Supporting Information** Additional supporting information can be found online in the Supporting Information section.

## Data Availability

Data sharing is not applicable to this article as no new data (except for serological data) were created or analyzed in this study.
