# Supplementary material for: Significant Impact of Schmallenberg Virus in Three Ruminant Farms: A Laboratory Experience
Source: Vet Med Int. 2026 Jan 5;2026:9979035. doi: 10.1155/vmi/9979035 (PMC12767771; doi:10.1155/vmi/9979035)
Supplement: Supplementary file 1 — Supporting Information Additional supporting information can be found online in the Supporting Information section. [file VMI-2026-9979035-s001.docx]

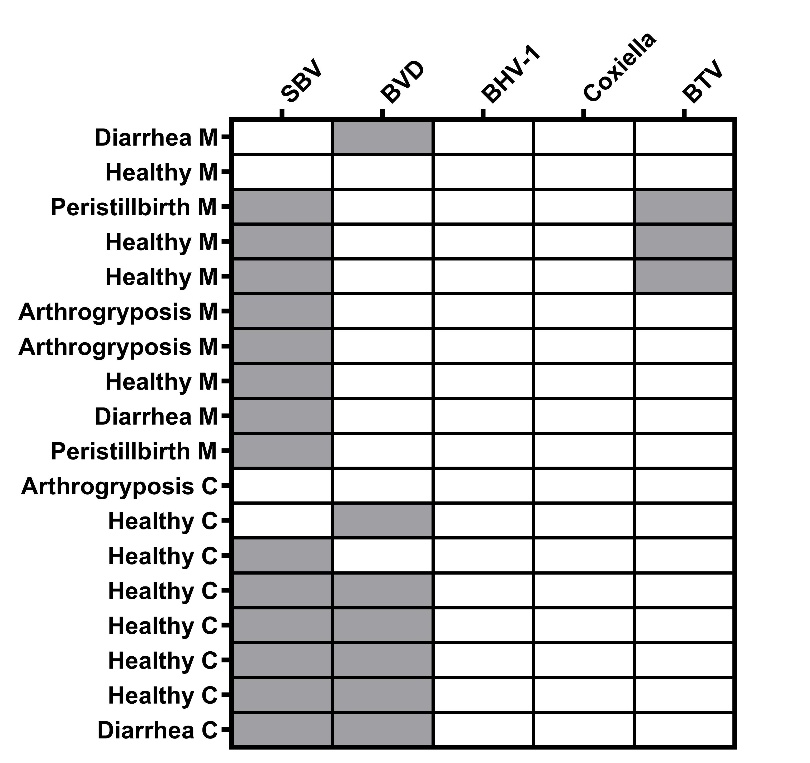


Farm 1: ELISA’s outcome obtained from the various serological tests (SBV, BVD, BHV-1, Coxiella burnetii, BTV) in farm n1. The grey color indicates a positive result, and the blank color indicates a negative result. Each sample has been identified based on symptoms (“healthy”, “diarrhea”, “stillbirth/perinatal mortality”, “arthrogryposis”) and age (“M” indicates “mother” and “C” indicates “calf”).


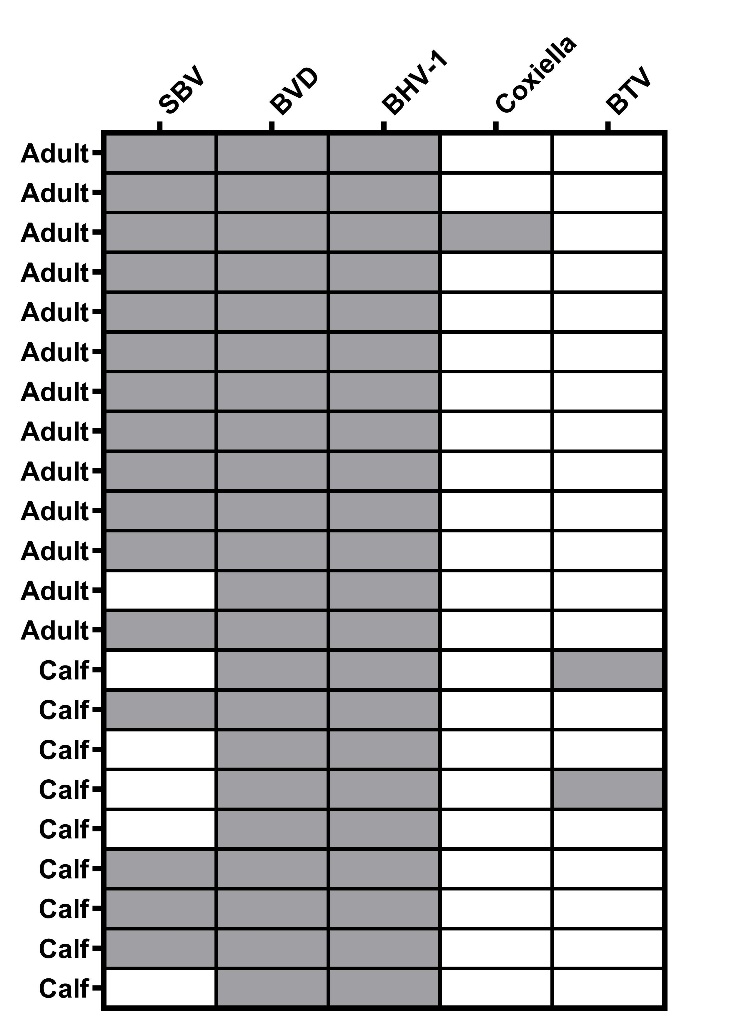


Farm 2: ELISA’s outcome obtained from the various serological tests (SBV, BVD, BHV-1, Coxiella burnetii, BTV) in farm n2. The grey color indicates a positive result, and the blank color indicates a negative result. Each sample has been identified based on age in adults and calves.


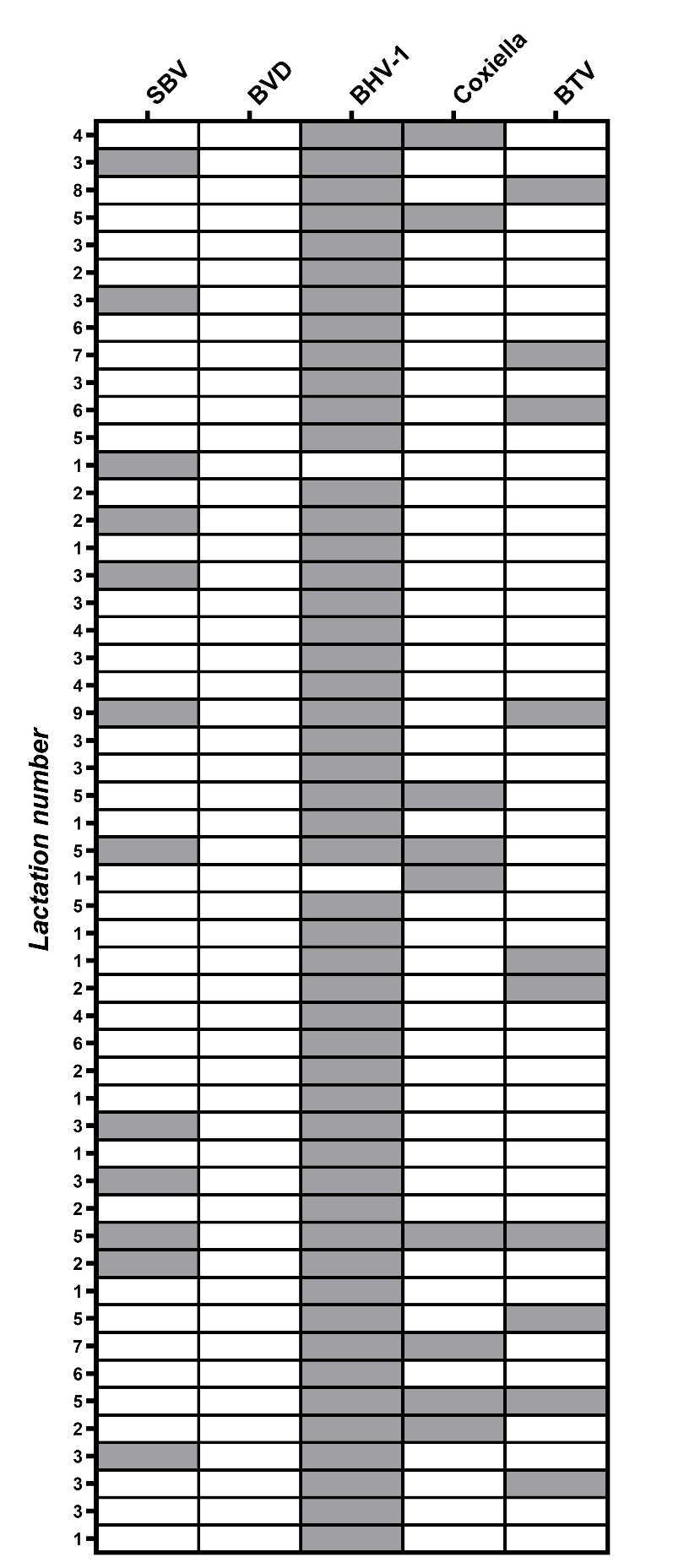


Farm 3: ELISA’s outcome obtained from the various serological tests (SBV, BVD, BHV-1, Coxiella burnetii, BTV) in farm n3. The grey color indicates a positive result, and the blank color indicates a negative result. Each sample has been identified based on the number of lactations.

|  | n | SBV | CI  % | *C. burnetii* | CI  % | BTV | CI  % | BVD* | CI  % | BHV-1* | CI  % |
| --- | --- | --- | --- | --- | --- | --- | --- | --- | --- | --- | --- |
| Factor |  | Positive (%) |  | Positive (%) |  | Positive (%) |  | Positive (%) |  | Positive (%) |  |
| Total | 92 | 42 (45.6) | 35.5−55.8 | 10 (10.8) | 4.5−17.2 | 15 (16.3) | 8.8−23.9 | 29 (31.5) | 22−41 | 72 (78.3) | 69.8−86.7 |
| Farm |  |  |  |  |  |  |  |  |  |  |  |
| 1 | 18 | 14 (77.8) | 58.6−97 | 0 (0) |  | 3 (16.7) | 0−33.9 | 7 (38.9) | 16.4−61.4 | 0 (0) |  |
| 2 | 22 | 16 (72.7) | 54.1−91.3 | 1 (4.5) | 0−13.2 | 2 (9) | 0−21.1 | 22 (100) |  | 22 (100) |  |
| 3 | 52 | 12 (23.1) | 11.6−34.5 | 9 (17.3) | 7−27.6 | 10 (19.2) | 8.5−29.9 | 0 (0) |  | 50 (96.1) | 90.9−100 |
| Age |  |  |  |  |  |  |  |  |  |  |  |
| Adults | 75 | 32 (42.7) | 31.5−53.9 | 2 (2.7) | 0−6.3 | 13 (17.3) | 8.8−25.9 | 14 (18.7) | 9.8−27.5 | 63 (84) | 75.7−92.3 |
| *Farm 1* | 10 | 8 (80) | 55.2−100 | 0 (0) |  | 3 (30) | 1.6−58.4 | 1 | 0−25.6 | 0 (0) |  |
| *Farm 2* | 13 | 12 (92) | 77.8−100 | 1 (7.7) | 0−22.2 | 0 (0) | − | 13 |  | 13 (100) |  |
| *Farm 3* | 52 | 12 (23.1) | 11.6−34.5 | 9 (17.3) | 7−27.6 | 10 (19.2) | 8.5−29.9 | 0 |  | 50 (96.1) | 90.9−100 |
| Calves | 17 | 10 (58.8) | 35.4−82.2 | 0 (0) |  | 2 (11.8) | 0−27.1 | 15 (88.2) | 72.9−100 | 9 (52.9) | 29.2−76.7 |
| *Farm 1* | 8 | 6 (75) |  | 0 (0) |  | 0 (0) |  | 6 (75) |  | 0 (0) |  |
| *Farm 2* | 9 | 4 (44.4) |  | 0 (0) |  | 2 (22.2) |  | 9 (100) |  | 9 (100) |  |
| *Farm 3* | 0 | 0 (0) |  | 0 (0) |  | 0 (0) |  | 0 (0) |  | 0 (0) |  |
| Lactation 1-4 | 33 | 9 (27.3) | 12.1−42.5 | 3 (9.1) | 0−18.9 | 3 (9.1) | 0−18.9 | 0 (0) |  | 31 (93.9) | 85.8−100 |
| Lactation 5-9 | 19 | 3 (15.8) | 0−32.2 | 6 (31.6) | 10.7−52.5 | 7 (36.8) | 15.1−58.5 | 0 (0) |  | 19 (100) |  |

CI= confidence interval 95%

Table 1 suppl.file: Data obtained from the analysis of three farms in southern Italy: complete results and confidence intervals

|  |  | SBV |  |  | *C. burnetii* |  |  | BTV |  |  |  |
| --- | --- | --- | --- | --- | --- | --- | --- | --- | --- | --- | --- |
| Factor | n | Positive | % | ***p*** | Positive | % | ***p*** | Positive | % | ***p*** |  |
| Total | 92 | 42 | 45.6 |  | 10 | 10.8 |  | 15 | 16.3 |  |  |
| Farm |  |  |  |  |  |  |  |  |  |  |  |
| Farm 1 | 18 | 14 | 77.8 |  | 0 | 0 |  | 3 | 16.7 |  |  |
| Farm 2 | 22 | 16 | 72.7 | **0.001** | 1 | 4.5 | 0.14 | 2 | 9 | **0.01** |  |
| Farm 3 | 52 | 12 | 23.1 |  | 9 | 17.3 |  | 10 | 19.2 |  |  |
| Age |  |  |  |  |  |  |  |  |  |  |  |
| Calves | 17 | 10 | 58.8 |  | 0 | 0 |  | 2 | 11.8 |  |  |
|  |  |  |  | 0.49 |  |  | 1 |  |  | 1 |  |
| Adult | 75 | 32 | 42.7 |  | 2 | 2.7 |  | 13 | 17.3 |  |  |
| Lactation |  |  |  |  |  |  |  |  |  |  |  |
| 1−4 | 33 | 9 | 27.3 |  | 3 | 9.1 |  | 3 | 9.1 |  |  |
|  |  |  |  | 0.5 |  |  | 0.14 |  |  | 0.07 |  |
| 5−9 | 19 | 3 | 15.8 |  | 6 | 31.6 |  | 7 | 36.8 |  |  |

Table 2 suppl.file: Statistical analysis (Fisher's exact test) of the results obtained
